# How patients being treated for non-small cell lung cancer value treatment benefit despite side effects

**DOI:** 10.1007/s11136-021-02882-6

**Published:** 2021-05-31

**Authors:** Mona L. Martin, Julia Correll, Andrew Walding, Anna Rydén

**Affiliations:** 1 Health Research Associates, Inc., Seattle, WA USA; 2 AstraZeneca R&D, Cambridge, UK; 3 AstraZeneca, Gothenburg, Sweden

**Keywords:** Interview studies, Non-small cell lung cancer, Qualitative, Side effects, Symptoms, Treatment benefit

## Abstract

**Purpose:**

To describe symptoms and side effects experienced by patients with advanced non-small cell lung cancer (NSCLC), assess how patients allocate sensations (i.e. symptoms or side effects) to either the disease or its treatment, and evaluate how patients balance side effects with treatment benefits.

**Methods:**

Qualitative sub-studies were conducted as part of two clinical trials in patients treated for advanced NSCLC (AURA [NCT01802632]; ARCTIC [NCT02352948]).

**Results:**

Interviews were conducted with 23 patients and 19 patients in the AURA and ARCTIC sub-studies, respectively. The most commonly experienced symptoms/side effects were respiratory (81% of patients), digestive (76%), pain and discomfort (76%), energy-related (71%), and sensory (62%). Patients identified a sensation as a treatment side effect if they had not experienced it before, if there was a temporal link between the sensation and receipt of treatment, and/or if their doctors consistently told or asked them about it in relation to side effects. Themes that emerged when patients talked about their cancer treatment and its side effects related to the serious nature of their advanced disease and their treatment expectations. Patients focused on treatment benefits, wanting a better quality of life, being hopeful, not really having a choice, and not thinking about side effects.

**Conclusions:**

In these two qualitative sub-studies, patients with advanced NSCLC valued the benefits of their treatment regardless of side effects that they experienced. Patients weighed their options against the seriousness of their disease and expressed their willingness to tolerate their side effects in return for receiving continued treatment benefits.

**Supplementary Information:**

The online version contains supplementary material available at 10.1007/s11136-021-02882-6.

## Introduction

Patients’ perception of their treatment experience contributes important information when assessing treatment benefit [1–4]. However, patients do not always fully report the range of symptoms they experience. Under-reporting can occur on the clinicians’ part too, as they process the subjective information that is provided by patients [2, 3]. Multiple studies have reported that physicians and nurses underestimate symptom onset, frequency, and severity in patients in comparison to patients’ ratings [5–8]. Knowing about patients’ treatment expectations, previous experience with side effects, current difficulties with tolerating side effects, and views of the balance between the benefits of treatment and the need to tolerate side effects, is important both to the drug development process and to regulatory agencies when considering the overall safety of newly developed compounds. This information is particularly relevant to new cancer therapies, which have diverse mechanisms of action, heterogeneous side effect profiles, and are often administered on a daily basis over prolonged treatment periods [9].

The standardized adverse event reporting process in clinical trials does not usually include patient perceptions of symptom severity and the degree of bother or difficulty caused by various symptoms. Without understanding how patients process the experience of symptoms and impacts, which can range from not-at-all to very severe, frequent, bothersome, or difficult-to-cope-with, it is hard to know how these aspects affect patients’ willingness to tolerate side effects in return for an actual or anticipated treatment benefit. Patient self-assessment of tolerability could potentially increase early withdrawal from clinical trials; however, it may also be a driver of more comprehensive reporting of side effects in trials, providing an enhanced understanding of the safety profile of a treatment. The US Food and Drug Administration Oncology Center of Excellence recently identified patient-focused drug development as an important program to advance cancer therapeutic development [9].

Information on symptoms and side effects was collected in two qualitative sub-studies of patients with non-small cell lung cancer (NSCLC): the AURA and the ARCTIC sub-studies. The AURA sub-study was conducted as part of the phase I/II AURA clinical trial, which assessed treatment for NSCLC with osimertinib, an epidermal growth factor receptor (EGFR) tyrosine kinase inhibitor (TKI) [3, 10]. The ARCTIC sub-study was conducted as part of the phase III ARCTIC clinical trial in patients with advanced NSCLC treated with durvalumab, a programmed cell death ligand 1 (PD-L1) antibody, and tremelimumab, an anti-cytotoxic T-lymphocyte-associated antigen 4 (CTLA-4) [11, 12].

Previous results from the AURA sub-study showed that some symptoms or side effects that were reported only by a small number of patients received high severity and bothersomeness scores, although patients rated their overall difficulty coping with side effects as low (mean difficulty with coping score: 2.0 [standard deviation: 2.3] out of a possible maximum of 10.0) [3]. It is not clear whether the small numbers of patients reporting the more severe and bothersome symptoms were insufficient to affect the overall difficulty with coping ratings, or whether something independent of the perceived severity and bothersome levels was driving how patients acted on their experiences of symptoms and side effects. Possibly there is something unique about how patients view side effects from cancer treatment compared with other types of treatments and other therapeutic conditions.

The current research uses data from the AURA and ARCTIC qualitative sub-studies to describe the symptoms and side effects experienced by patients with advanced NSCLC, to assess how patients allocate sensations (i.e. symptoms or side effects) to either the disease or its treatment, and to evaluate how patients balance side effects with treatment benefits. Qualitative interview data were collected in both sub-studies to provide a better understanding of the patient-perceived experience. The interview questions were similar across the two studies, allowing the data from the AURA and ARCTIC sub-studies to be combined to increase the sample size and the amount of information available for exploring patient perceptions of side effects and treatment benefit.

## Methods

### Study design and participants

The current report presents results from the phase I/II AURA and phase III ARCTIC trial qualitative sub-studies. The qualitative analysis used in this research was based on similarity of content, where coding and theme development explicitly reflected the content of the interview (semantic type of thematic analysis) [13–15]. The theoretical framework was a qualitative content analysis and an inductive approach was used to develop concept codes.

### AURA

AURA was an open-label dose-escalation and dose-expansion study of osimertinib in adult patients with locally advanced or metastatic NSCLC, who had EGFR-TKI-sensitizing mutations or who had received prior clinical benefit from EGFR-TKI treatment, and who had disease progression while receiving previous treatment with an EGFR-TKI (ClinicalTrials.gov ID: NCT01802632) [10]. The study was conducted across seven sites in the USA, UK, Spain, and South Korea. Patients who received the study drug were invited to participate in the qualitative sub-study [3].

### ARCTIC

ARCTIC was a randomized, open-label study in adult patients with locally advanced or metastatic NSCLC, who did not have EGFR tyrosine kinase-activating mutations or anaplastic lymphoma kinase rearrangements, and who had received at least two prior systemic anti-cancer regimens, including one platinum-based chemotherapy (NCT02352948) [11, 12]. ARCTIC assessed the clinical activity and safety of durvalumab versus standard of care (erlotinib, gemcitabine, or vinorelbine) in patients with ≥25% of tumor cells (TCs) expressing PD-L1, and the combination of durvalumab and tremelimumab versus standard of care in patients with <25% of TCs expressing PD-L1 [11]. The study was conducted in 26 countries across North America, Latin America, Asia, and Europe. Patients who were interested in participating in the sub-study were identified during the clinical trial recruitment and enrollment procedures at 14 participating sites across the USA, UK, and Canada.

### Interviews

Study coordinators invited all patients being enrolled in the AURA and ARCTIC clinical trials to participate in the qualitative interviews. All patients who accepted participation were interviewed. Patients declined participation if they felt too ill or had competing concerns about disease progression and had other overriding concerns and needs. All patients who accepted participation were interviewed. No tracking was done regarding responder status from the clinical trial data as it did not directly relate to the purpose of the qualitative interviews.

For the current qualitative analyses, data were obtained from telephone interviews conducted approximately 4 to 6 weeks after the initiation of study treatment. Each individual interview lasted approximately 30 min. During the interviews, patients were asked to describe a typical day before and during clinical trial participation, and how they experienced symptoms of NSCLC at these two time points. Patients were first asked to describe their symptoms (spontaneously and then probed), and were then asked to describe what the symptom feels like, the frequency and duration of the symptom, symptom severity, and how bothersome the symptom is. Follow-up interviews (after approximately 4 months of treatment), which were conducted to detect any longitudinal differences, do not form part of the current report. In each of the two studies, four different interviewers conducted the qualitative interviews. The interviewers were experienced in qualitative interview techniques and had received training on how to use the interview guides through a process of mock interview sessions.

**Fig. 1:**
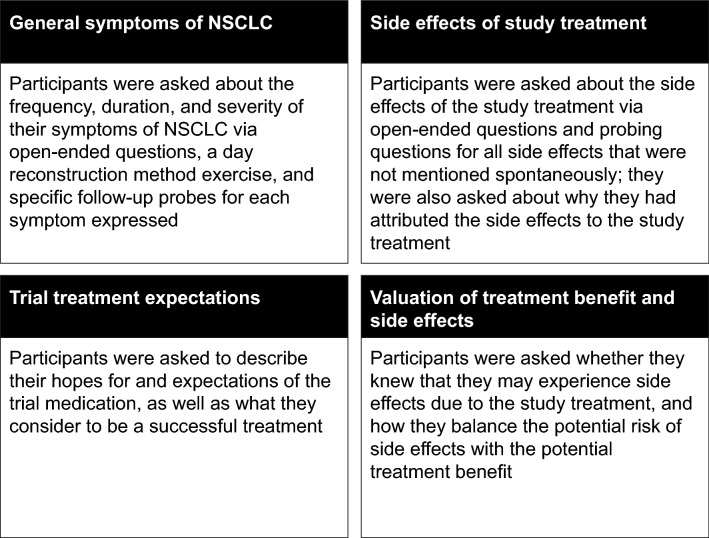
Key topics in interview guide. *NSCLC* non-small cell lung cancer

Semi-structured interview guides were used to focus patients on the symptoms and side effects that they had experienced before trial entry, as well as those that they experienced during the trial. Patients were also asked to discuss their expectations of their current treatment, what they thought of the balance between the risks and the benefits of their current treatment, and any thoughts they had about how they defined treatment success. Figure 1 provides an overview of the content covered in the interview guides.

### Qualitative analysis

All interviews were audio-recorded and transcribed for analysis. For non-English language interviews, recordings in the AURA sub-study were first transcribed and then translated into English, whereas in the ARCTIC sub-study interviews were simultaneously translated into English for coding. To group and organize quotations that had similar content, transcripts were coded using a coding framework and ATLAS.ti™ software (versions 7.0 and 7.5 in the AURA and ARCTIC sub-studies, respectively; ATLAS.ti Scientific Software Development GmbH, Berlin, Germany).

Data quality was evaluated for data completeness, and for consistency of assigned codes. Seven transcripts were independently dual-coded and assessed for inter-coder agreement. Three coders were used for this study and consistency of coding was characterized by the overall percent of agreement in the identification of concepts, and the assignment of codes to each identified concept. Saturation of concept was evaluated by ordering the transcripts chronologically and then creating groups of five or six transcripts each. Concepts in the first transcript group were coded, and then the newly appearing codes from each subsequent group of transcripts were compared with the codes established in previous groups to assess whether any new concept codes had arisen. Saturation of concept was considered to be reached when no new concepts appeared [16].

### Ethics approval

The AURA and ARCTIC trials and sub-studies were performed in accordance with the ethical principles that have their origin in the Declaration of Helsinki and were consistent with Good Clinical Practice guidelines and the applicable regulatory requirements. The trials and sub-studies were approved by the Institutional Review Boards or independent Ethics Committees (listed in full in the appendix). All data collected were handled as strictly confidential in accordance with local, state, and federal laws. The AURA and ARCTIC sub-studies posed no known risks to the patients.

## Results

### Patients and data quality

Interviews were conducted with 23 patients in the AURA sub-study and with 19 patients in the ARCTIC sub-study. All patients in the AURA sub-study were being treated with osimertinib. At the first interview in the ARCTIC sub-study, 10 patients were receiving treatment with durvalumab, eight with durvalumab plus tremelimumab, and one with standard of care. The median age in the AURA sub-study was 62 years (range 33–82 years), and 35% were male [3]. In the ARCTIC sub-study, the median age was 66 years (range 44–70 years), and 58% were male.

In the AURA sub-study, no new concepts appeared after the third of four groups of transcripts, and agreement of codes assigned ranged from 92.0% to 95.5% between coders [3]. In the ARCTIC sub-study, no new concepts appeared after the third of four groups of transcripts, and agreement on the assignment of concept codes ranged from 90.5% to 100.0% among the three coders.

### Predominance of concept expressions

Table 1 shows the numbers and proportions of patient expressions and of the interview transcripts that contributed to each of the symptom or side effect concepts in the AURA and ARCTIC sub-studies. The predominance of an expression was a measure of how much it was talked about by patients compared with everything else that they mentioned. The percentage of individual patient interview transcripts that contributed a coded concept indicated how many different patients contributed to that concept.

**Table 1:** Coded data frequency for symptom or side effect concepts

	AURA sub-study (*N* = 23)		ARCTIC sub-study (*N* = 19)	
Symptoms or side effects	*n* (%) of 662 language expressions	*n* (%) of 23 transcripts	*n* (%) of 359 language expressions	*n* (%) of 19 transcripts
Energy-related^a^	85 (13)	15 (65)	60 (17)	15 (79)
Pain and discomfort^b^	105 (16)	15 (65)	67 (19)	17 (90)
Respiratory^c^	132 (20)	18 (78)	83 (23)	16 (84)
Digestive^d^	90 (14)	16 (70)	59 (16)	16 (84)
Sleep disturbances^e^	6 (1)	5 (22)	12 (3)	8 (42)
Oral^f^	33 (5)	9 (39)	8 (2)	6 (37)
Skin and nail^g^	140 (21)	17 (74)	25 (7)	8 (42)
Sensory^h^	34 (5)	11 (48)	23 (6)	12 (63)
Genitourinary^i^	1 (0)	1 (4)	0 (0)	1 (5)
Additional^j^	36 (5)	8 (35)	22 (6)	11 (58)

^a^Includes exhaustion, fatigue, low energy, low stamina, tiredness, and weakness

^b^Includes abdominal pain, achiness, back pain, chest pain, eye pain, headache, fibromyalgia, hip-waist pain, joint or bone pain, mouth pain, muscle pain or cramping, neck pain, nipple pain, pain (unspecified), pain in extremities, side pain, shoulder pain, sore or painful skin, stiffness, and whole-body pain

^c^Includes bronchitis, coughing, coughing-up blood, difficulty breathing, dry nose, fluid in lungs, phlegm or mucus, pneumonia, runny nose or cold, shortness of breath, and stridor or wheezing

^d^Includes black stool, bowel incontinence, constipation, diarrhea, difficulty swallowing, full stomach, gas, heartburn or acid reflux, heaving, nausea, poor appetite, vomiting, weight gain, and weight loss

^e^Includes difficulty falling asleep, difficulty staying asleep, excessive sleep, nightmares, and reduced sleep quality

^f^Includes coarse tongue, dry mouth, dry throat, hoarseness, mouth sores, sore gums, sore throat, vocal cord paralysis, and voice changes

^g^Includes acne, burning, dry nails, dry skin, hair growth, itching, peeling, rash, sensitive skin, skin discoloration, and sores

^h^Includes changes in taste, chills, dizziness, fainting, feels hot, fever, hot flashes, numbness or tingling, and vision difficulties

^i^Includes frequent urinary tract infections and urinary odor

^j^Includes cognitive problems, confusion, difficulty concentrating, dry eyes, hyperthyroid, low blood pressure, memory loss, menstrual changes, mentally tired, night sweats, puffy eyes, shaky, swelling, and swollen lymph nodes

In each of the two sub-studies, more than half of the patients talked about experiencing respiratory (81% of patients overall), digestive (76%), pain and discomfort (76%), energy-related (71%), and sensory (62%) symptoms or side effects. There were no marked differences between the ARCTIC and the AURA sub-studies regarding the proportions of transcripts contributing to each of the concept expressions (Table 1). However, for most concept expressions, the proportion of patients contributing was larger in the ARCTIC sub-study than in the AURA sub-study. The largest absolute percentage difference between the two study groups was for skin and nail-related symptoms or side effects, which 74% of patients in the AURA sub-study spoke about compared with 42% of patients in the ARCTIC sub-study. These differences reflected safety data from the two trials, which showed a relatively high incidence (>20%) of rash and nail-related side effects with osimertinib in the AURA study, whereas such side effects were not as commonly seen in the ARCTIC study.

### What were patients’ expectations regarding clinical trial enrollment and successful treatment?

When patients were asked during the qualitative interview process to describe what it was that they hoped to attain by participating in the clinical trial, the responses in the two sub-studies fell into four main themes, covering a range of desires: ‘hope to maintain or regain quality of life,’ ‘hope that the cancer shrinks or stops growing,’ ‘hope for a longer life,’ and ‘hope for a cure.’ Examples of quotations from each of the themes are shown in Table 2.

**Table 2:** Patients’ expectations of clinical trial participation: common themes

Theme	AURA sub-study example quotations^a^	ARCTIC sub-study example quotations^a^
Hope to maintain or regain quality of life	• “I don’t know what’s going to happen. I’m expectative, and hopeful … I hope to be able to tell you [later] that my symptoms are absolutely tolerable, I’m accustomed to them, and my life is absolutely normal.” • “[I expect the treatment to] improve my breathing. Improve my feeling of well-being … [I expect] generally to feel a bit better.” • “It’s just good as it is now … the fact that it makes your daily routine possible … It would be a good treatment if it allows you to do your normal activities and to keep your job.”	• “I’m hoping it’s gonna work, is that I can build myself to be strong enough to live a standard way of life that I used to, as far as being able to work and able to not have to sleep as much.” • “I just want to live normally … I know my time is limited … I want a good quality of life, I want to be able to work, I want to be able to be coherent and know what’s going on.” • “I’m hoping that it will do me more quality of years left, I’m hoping to get another, it’d be nice if I get another 10 years.”
Hope that the cancer shrinks or stops growing	• “There will be a time when I develop tolerance to the treatment. Before the development of tolerance, I really wish that it will reduce my tumor and cancer cells a lot.” • “[I expect to] stop the spread of cancer. Contain it maybe is a better word.” • “My expectations, in [the] beginning [were] that it would destroy all the … cancer. Now my expectations are that it will keep the cancer under control.”	• “[I hope this drug will] shrink the tumor; I wasn’t going for a cure.” • “I hope that it doesn’t spread, and I hope that it reduces the tumors.” • “I don’t think there’s going to be a magical cure, but I think if it can shrink that tumor a bit, give me a bit more time … then I’ll be quite happy.”
Hope for a longer life	• “Well, my expectations are that … I’ll stay like this with the illness contained, for the longest period of time possible … All of the years I can add to this life are welcome.”	• “I am hoping for a miracle, but you know they don’t happen very often … I hope it can extend my life, if I’m being realistic.” • “I’d like the opportunity to try [the study treatment] and see if it will give me a remarkable result, because anything that keeps me alive a bit longer is probably quite good.”
Hope for a cure	• “After I started this treatment, my lump size is reduced so much which gave me some expectations of cure.” • “I wish it could continuously help me get better and it will cure my lung cancer eventually.” • “I hope I could get more size reduction [of tumor] with which I can expect a complete cure of cancer at the end.”	• “Obviously, everybody always hopes for an actual cure.” • “What was I hoping [this treatment] would do? Kill the cancer.” • “I thought, well maybe the job of this [treatment] stuff is to get rid of [the cancer].”

^a^Bracketed text indicates coder additions to clarify context

Patients were asked during the interview to describe what they would consider to be a successful treatment, in order to explore how patients thought about and recognized treatment benefits. The descriptions were broadly similar to those of the desired outcomes of trial participation and fell into three main themes: ‘treatment that provides improved quality of life,’ ‘treatment that makes cancer shrink or stop growing,’ and ‘treatment that cures cancer.’ Example quotations are listed in Table 3.

**Table 3:** Patient descriptions of a “successful treatment”

Theme	AURA sub-study example quotations^a^	ARCTIC sub-study example quotations^a^
Treatment that provides improved quality of life	• “A treatment that … will eventually help many others from suffering [is successful].” • “The fact that … I’m able to live a normal life is the great benefit … [I] consider this a successful treatment.” • “A successful treatment at this point … allows me to do my daily activities.”	• “My overall life, what I can do on a daily basis. I want to be able to have treatment and still be able to have some kind of a life. Where the side effects will not be limiting me.” • “It’s already successful … I’ve gotten about an increase of 60% to 70% in my energy.”
Treatment that makes cancer shrink or stop growing	• “A successful treatment will make your cancer cells reduced and bring you hope in a complete cure.” • “So, a successful treatment is I take these pills every day, and providing I keep taking them, the cancer doesn’t go up, it stays under control.”	• “If it’ll slow that cancer down or keep from growing, well I’d say that’d make it successful.” • “[A successful treatment is] one that could like make the cancer dormant, stop growing, or make it totally go away.”
Treatment that cures cancer	• “A successful treatment means cure.” • ﻿“I [consider] the chances of the cancer going [away].”	• “It’ll be wonderful if it does show that [the treatment] got rid of [the cancer].” • “[A successful treatment makes] this cancer probably disappear.”

^a^Bracketed text indicates coder additions to clarify context

### How do patients identify side effects of treatment?

While exploring what patients took into consideration when asked to allocate a sensation (i.e. a symptom or side effect) to either the disease or its treatment, three main themes arose showing how patients came to their decisions: ‘never experienced before,’ ‘timing of side effect,’ and ‘told by doctor it was a possibility.’ Examples of patient explanations of these three themes are presented in Table 4. The first of these themes was about patients recognizing a sensation surfacing that they had not experienced before, and the second theme concentrated on identifying a temporal link between the sensation and the receipt of treatment. Generally, as treatment events became repetitive, and the experience of symptom or side effect sensations fell into similar repetition, it was not difficult for patients to allocate these sensations to the treatment. Finally, the most conclusive support for allocating a sensation to either the disease or its treatment came from the medical community itself. In this regard, patients’ expressions ranged from considering what their doctors consistently asked them about when monitoring treatment tolerance and what their doctors specifically told them regarding what side effects they could expect.

**Table 4:** Patient allocation of sensations to symptoms versus side effects

Theme	AURA sub-study example quotations^a^	ARCTIC sub-study example quotations^a^
Never experienced before	• “I don’t normally have that. I didn’t have any acne before and I don’t, diarrhea was nonexistent.” • “I’ve always had skin that tended to be oily … From then on I had skin that was really dry, dehydrated, peeling, so evidently I had to associate that with the medication, I only attribute to the drug, because I didn’t have it before.”	• “The fatigue is definitely [a side effect]. I didn’t have that before.” • “Well I never had shortness of breath before like that. And this rash I never had that type of rash before.” • “It’s always like a day after where I do suffer a loose bowel I notice … which I never did before.”
Timing of side effect	• “I didn’t have any [hot flashes] while I was off the drug. But since I came back on them again, I’m starting to get them.” • “[I didn’t think rash was a symptom because] when they halved the dose, I felt pretty good and without any consequences.” • “Actually [I had] diarrhea twice that day after I’d taken the drug.”	• “[I attribute fatigue and constipation to treatment] because it started shortly after, as soon as I started getting treatment.” • “Anything I seem to get seems to come about three or four days after the treatment [itching, numb toes, joint pain].” • “[I attribute rash and itching to treatment because] it was the timing. After treatment … I wanted to itch every part of my body.”
Told by doctor it was a possibility	• “At first I didn’t know [if wrinkled fingertips] was a symptom, but … I asked whether it was a side effect and was told that it was.” • “I had mentioned it to my doctor and was told that [heat sensitivity and nail pain] are side effects from the medication.” • “I actually asked about this, and they said my hair will grow again if I stopped the medication.”	• “[I attribute itching and rash to treatment] probably because I was told that I may get it.” • “[My itchy rash] started about 2 weeks after the first treatment, and when I talked to the doctor about it he said, yes, that what you’re experiencing is one of the side effects.” • “Every time I go to the doctor, they question me and that always comes up, do I have any rashes … I’d think well it might be part of the side effects if they asked me that.”

^a^Bracketed text indicates coder additions to clarify context

A review of the transcript database showed occasional expressions of uncertainty about whether a sensation experienced should be allocated to the cancer or to its treatment. In these instances, the surrounding text in the transcript provided further context for the uncertainty, describing multiple treatments happening or *“so much going on”* in general. Some patients had previous experience with a particular symptom and were not sure whether it was continuing as a part of cancer progression or whether it was also related to the treatment. For some symptoms, patients were uncertain whether these were related to their cancer and its treatment or whether they were simply caused by age-related bodily changes.

### How do patients balance the experience of side effects of treatment with their desire for treatment benefit?

Five themes emerged across the two sub-studies when patients talked about their cancer treatment and its side effects: ‘focus on the fact that the treatment works,’ ‘want a better quality of life,’ ‘always hopeful,’ ‘don’t really have a choice,’ and ‘don’t think about side effects.’ Example quotations are presented in Table 5.

**Table 5:** Themes behind the balances between risk of side effects and treatment benefit

Theme	AURA sub-study example quotations^a^	ARCTIC sub-study example quotations^a^
Focus on the fact that the treatment works	• “The side effects are very minimal so as long as the treatment is going to take care or stop this cancer, it’s no question what’s the best deal.” • “[When thinking about treatment success, I consider] progress of the shrinkage in the tumor.” • “If it continues to recede… obviously I’m going to put up with all the symptoms and side effects I get in favor of having to get rid of the cancer.” • “Because my doctor said that I’m getting better and that the size of my cancer reduced, I would take this treatment again.”	• “It’s helping me compared to what I had before, it’s well worth it.” • “If it’s doing me some good, then these side effects don’t mean anything.” • “I’ll go for the treatment, successful treatment. I don’t care if I am in pain.” • “To me, it’s worth it [trying the study treatment] … To find out if it works.”
Want a better quality of life	• “[Good treatment is if] I can be with my family, in my house, and even though I have some discomforts, I can get used to them.” • “[I consider] quality of life, [my] ability to do things.” • “Capability of performing normal life activities would be [one of] my considerations.”	• “I wish I were a little more physically capable, I want to eat again and gain my weight back …” • “…the least side effects I can have and still function, no matter what they are, I want to be normal.” • “I wanted to have treatment and still be able to have some kind of life.”
Always hopeful	• “I think the treatment is really good. I feel great physically and mentally … I can only think of the benefits of the treatment.” • “I don’t think of the negative. I just think of the positive and I’m happy with it.”	• “I don’t know if it’s in the cards but we will keep trying what we can.” • “I guess I continue for the hope.” • “You know there is going to be side effects, so you are hoping it’s gonna help.”
Don’t really have a choice	• “[It’s] a terminal [illness] and quite a big illness … I didn’t feel like I had a lot choice [regarding treatment] … there’s not hesitation.” • “I would want to continue with the current treatment. And there are no other treatment options available for me.” • “I really don’t have a choice at this point, so I can’t complain about the small things now … the side effects are okay if you weigh the pros and cons.”	• “You don’t have a choice [about side effects]. The only choice is you’re not living very long if you don’t take up the trial.” • “I suppose the alternative is death, you are between a rock and a hard place.” • “I felt I had nothing to lose.” • “I didn’t have any options, this was my last opportunity to try something new, so I didn’t worry about it.”
Don’t think about side effects	• “[Side effects are] not a problem. I am willing to go along with these little side effects as long as it is working and keeping me going.”	• “I’m quite a positive person, I don’t look at the down side of things.” • “I’m not the least bit negative, so side effects was the least of my worries.” • “I didn’t think a thing about the side effects, I didn’t pay attention to it at all.”

^a^Bracketed text indicates coder additions to clarify context

The wider context expressed when patients talked about their thoughts regarding treatment and side effects tended to be related to the dire nature of their situation (e.g. *“I suppose the alternative is death, you are between a rock and a hard place.”*) and what they were looking for in a treatment. Patients talked about tolerating their side effects in return for treatment benefit, and many expressed a hopeful attitude. Several patient expressions demonstrated the endeavor to balance the negative impact of side effects against the sense that the treatment was helping (e.g. *“If it continues to recede … obviously I’m going to put up with all the symptoms and side effects I get in favor of having to get rid of the cancer.”*). Some patients did not allow themselves to think about any negative aspects of treatment (e.g. *“I don’t think of the negative. I just think of the positive and I’m happy with it.”*). Previous experiences were considered, showing that patients evaluated the side effects that they were currently experiencing against those that they had experienced in the past, and previous difficulty with side effects was a possible deterrent to considering new treatments.

Patients expressed themes around not having any choice except to tolerate side effects if they wanted to live longer, and having no hesitation regarding treatment. Patients commented that their advanced NSCLC was *“a terminal [illness] and quite a big illness …”*, that *“there are no other treatment options available for me”* and that the *“only choice is you’re not living very long if you don’t take up the trial.”* One patient said that *“I can’t complain about the small things now … the side effects are okay if you weigh the pros and cons.”*

## Discussion

The current article reports results from two qualitative sub-studies conducted as part of the phase I/II AURA and the phase III ARCTIC clinical trials. In the two sub-studies, semi-structured interviews were used to elicit qualitative data on symptoms, side effects, and expectations from patients receiving treatment for NSCLC in one of two clinical trials. The aims were to describe the symptom or side effect sensations experienced by patients with NSCLC and to explore patients’ expectations about clinical trial participation, and how patients balance treatment benefits and side effects.

Patients participating in the AURA sub-study or the ARCTIC sub-study were interviewed approximately 4 to 6 weeks after the initiation of their study treatment. This time frame allowed patients to become used to their new treatment and to notice any changes in the pattern of their symptom or side effect sensations. The trials both enrolled patients with advanced NSCLC, but they assessed different targeted therapies. In the AURA sub-study, patients received oral anti-cancer therapy with the EGFR-TKI osimertinib after having experienced progression of their cancer during previous EGFR-TKI therapy [10]. Patients in the ARCTIC sub-study received intravenous anti-cancer therapy with durvalumab, durvalumab plus tremelimumab, or standard of care (with erlotinib, gemcitabine, or vinorelbine) after having received at least two previous systemic treatments for their cancer, including chemotherapy [11, 12]. Thus, patients in the ARCTIC sub-study had received more prior lines of treatment than patients in the AURA sub-study. The patterns of symptom or side effect concepts mentioned by patients in the AURA and in the ARCTIC sub-studies were generally similar, with most patients in both sub-studies talking about respiratory, digestive, and energy-related concepts, as well as pain and discomfort. A higher proportion of patients in the AURA sub-study than in the ARCTIC sub-study spoke about nail-related issues, which is in line with the known profile of osimertinib, which was the treatment assessed in the AURA trial.

Demonstrated improved quality of life and reduced toxicity are particularly important when assessing the evidence of clinical benefit from new oncology treatments in the non-curative setting [17]. Themes that arose from the sub-studies showed that patients were hopeful that their anti-cancer treatment would work, and that they were willing to tolerate treatment side effects in return for treatment benefits and a better quality of life. Not focusing on the side effects and not really having a choice but to tolerate side effects were also themes. The current results suggest that the dire nature of their cancer and the hope of a treatment benefit drove how patients acted on symptom and side effect experiences, including whether or not they reported them. Previous results from the AURA sub-study showed that, although some symptoms or side effects rated by only a few patients were scored as highly severe and highly bothersome, overall patients’ difficulty coping with side effects was rated as low [3]. It appears from the current results of the two sub-studies that patients with advanced cancer cope with the perceived severity and bothersome levels of their experiences by placing them into a wider context (e.g. *“If it continues to recede … obviously I’m going to put up with all the symptoms and side effects I get in favor of having to get rid of the cancer.”*). It may be that patients are more willing to cope with the side effects of anti-cancer treatments than with those relating to treatment for other, more benign conditions.

Separating symptoms of a condition from the side effects of a treatment is a difficult task to ask of patients [18], and can be particularly challenging in oncology because sensations can relate to both. While it is generally believed that patients cannot correctly attribute the sensations that they experience to the disease versus its treatment because it is thought that patients do not understand the underlying clinical aspects, pathology, or pharmacology, themes that arose from the current sub-studies suggest that patients were fairly capable of distinguishing between the symptoms of their disease and the side effects of treatment. Past experience and education received from their medical team provided an important reference when patients made decisions about symptom or side effect attribution. Patients assessed whether the sensation was a new experience, in which case there was an increased likelihood that it was a treatment-related sensation. Although it is possible that new symptoms arise that are linked to the progression of cancer, this differentiation on the part of the patient presents a good foundation for sorting where the symptoms belong, particularly when combined with a temporal link between receiving a treatment and experiencing a sensation. Sensations that had been mentioned by the treating physician as potential side effects of treatment were remembered by patients, and attributed that way, which underscores the importance of physicians communicating information on potential side effects prior to the start of treatment. Results from this study demonstrate how additional information could be elicited from patients in treatment trials regarding their side effects; for example, by asking specifically about whether any new sensations have arisen since the start of the new treatment. Clinical trials may also benefit from collecting and presenting symptom or side effect information longitudinally over time from treatment initiation, to show any change in patterns over the course of the treatment and follow-up periods.

In the current sub-studies, patients’ expectations regarding their clinical trial enrollment were similar in content to their descriptions of what they thought the characteristics of a successful treatment would be. Patients spoke about wanting to maintain, regain, or improve their quality of life and wanting the cancer to shrink or to stop growing. These expectations are reflected in the results of another qualitative study of patients considering participation in early phase oncology clinical trials, the large majority of whom were motivated by potential clinical benefit and about half of whom expected tumor shrinkage [19].

Saturation results suggest that continued interviews would not be likely to provide any further new information, and the interview results obtained were sufficient to elicit a full picture of the concepts important to this patient population. The sample size in each of the two sub-studies (23 and 19 patients in the AURA and the ARCTIC sub-studies, respectively) was typical for qualitative interview studies [18, 20, 21]. Combining the data from the AURA and ARCTIC sub-studies increased sample size and information available for exploring and comparing patient perceptions about the balance between tolerating treatment side effects and the desire for a treatment benefit. Further, the assessment of inter-rater agreement was used to evaluate the consistency of the assignment of codes to assure the different coders were processing the interview data in the same way. A total of seven transcripts were dual-coded and compared for agreement between coders in the assignment of codes to transcript text. Ideally, there should be over 90% agreement [15]. The results of this evaluation ranged from 90.5% to 100.0% for the different pairs of coders that were used.

A potential limitation of these sub-studies is that interview participants were enrolled in a clinical trial and thus may have had less comorbidity (or slightly different mixes of comorbidity) than patients treated for advanced NSCLC in the real-world patient care settings. The AURA and ARCTIC trials both excluded patients with severe or uncontrolled systemic diseases, including active bleeding diatheses or active infection. In addition, all interviewed patients had metastatic disease, and most were receiving treatment with either osimertinib or durvalumab. Also, in reference to some of the suggested consolidated criteria for reporting qualitative research (COREQ): being part of a clinical trial and under strict study protocols regarding assessments and timings, patients were not re-interviewed to gain their reflections on the thematic results of the first interviews. Finally, while the quotations provided in this report are generally from separate individuals, there are some cases where a single participant might have contributed more than one statement to the sample quotations provided. Being sourced from two different sub-studies makes it difficult to identify by individual ID numbers those cases for this manuscript. These aspects should be taken into account when considering the generalizability of this research to the broader patient population with NSCLC.

## Conclusions

Qualitative results from the AURA and the ARCTIC sub-studies showed generally similar patterns of symptoms and side effects experienced by patients. Most patients described having pain and discomfort, as well as respiratory, digestive, and energy-related difficulties. These two sub-studies revealed a number of different influences that can affect how patients perceive and tolerate cancer treatment and its side effects. Patients identified a sensation as a treatment side effect if it had not been previously experienced, if it was temporally linked to the receipt of treatment, and/or if it had been specifically asked about or mentioned as a known side effect by the treating physician. While some patients preferred as their top priority treatments that would allow them to maintain aspects of quality of life and function, most patients expressed themes around the serious nature of having advanced NSCLC and about being willing to tolerate the negative impact of side effects to obtain treatment benefit.

## Supplementary Information

Below is the link to the electronic supplementary material.

Supplementary file1 (DOCX 40 kb)

Supplementary file2 (DOCX 43 kb)

## Data Availability

The data supporting the findings are available within the article.
