# Supplementary material for: How patients being treated for non-small cell lung cancer value treatment benefit despite side effects
Source: Qual Life Res. 2021 May 31;31(1):135–46. doi: 10.1007/s11136-021-02882-6 (PMC8800875; doi:10.1007/s11136-021-02882-6)
Supplement: Supplementary file 1 — Supplementary file1 (DOCX 40 kb) [file 11136_2021_2882_MOESM1_ESM.docx]

Appendix I

AURA Study: Independent Ethics Committees/Institutional Review Boards

Australia

- Austin Health Human Research Ethics Committee, Heidelberg, Australia
- Bellberry Human Research Ethics Committee, Dulwich, Australia

France

- Comite de Protection des Personnes ile de France VII, Le Kremlin-Bicetre, France

Germany

- Ethikkommission der Medizinischen Fakultät, der Universität zu Köln, Köln, Germany
- Ethik-Kommission der Universität Duisburg Essen, Essen, Germany
- Ethikkommission der Medizinischen Fakultät der Universität Würzburg, Würzburg, Germany

Italy

- EC AZ. Ospedaliera San Luigi Gonzaga, Orbassano, Italy
- Genova-Comitato Etico Liguria, Azienda Ospedaliera Universitaria San Martino, Genova, Italy
- Comitato Etico Degli Istiuti Fisioterapici Ospitalieri, Roma, Italy

Japan

- National Cancer Center Hospital East, IRB, Kashiwa-shi, Japan
- National Hospital Organisation Shikoku Cancer Center, IRB, Matsuyama-shi, Japan
- Osaka Medical College Hospital, IRB, Takatsuki-shi, Japan
- Kansai Medical University, Hirakata Hospital, IRB, Hirakata-shi, Japan
- Shizuoka Cancer Center, IRB, Sunto-gun, Japan
- Institutional Review Board of Chiba Cancer Center, Chiba, Japan
- Tokyo Medical University Hospital, IRB, Tokyo, Japan
- Kanazawa University Hospital, IRB, Ishikawa, Japan
- Osaka Prefectural Medical Center for Respiratory and Allergic Diseases, IRB, Osaka, Japan
- Institutional Review Board of Institute of Biomedical Research and Innovation Hospital, Hyogo, Japan
- Okayama University Hospital, IRB, Okayama, Japan
- Kyushu University Hospital, IRB, Fukuoka, Japan
- Yokohama Municipal Citizen’s Hospital, IRB, Yokohama Kanagawa, Japan
- Hiroshima City Hiroshima Citizens Hospital, IRB, Hiroshima, Japan

Republic of China

- Research Ethics Committee, National Taiwan University Hosp, Taipei, Taiwan, Republic of China
- Human Experiment & Ethics Committee, Tainan, Taiwan, Republic of China

South Korea

- Asan Medical Center (EC), Institutional Review Board, Seoul, South Korea
- Seoul National University Hospital (EC), Institutional Review Board, Seoul, South Korea
- Samsung Medical Center EC, Seoul, South Korea
- IRB of Severance Hospital, Yonsei University Health System, Seoul, South Korea

Spain

- Hospital Universitario Vall d´Hebrón IEC, Comité Ético de Investigación Clínica, Barcelona, Spain

United Kingdom

- North West – GM Central, Manchester, United Kingdom

United States of America

- Dana Farber Cancer Institute, Office Human Research Studies, Boston, MA, USA
- The University of Texas MD Anderson Cancer Centre, Institutional Review Board, Houston, TX, USA
- Western Institutional Review Board, Puyallup, WA, USA
- Vanderbilt University Institutional Review Board, Nashville, TN, USA
- Emory University IRB, Atlanta, GA, USA
- Chesapeake IRB, Columbia, MD, USA
