# Supplementary material for: How patients being treated for non-small cell lung cancer value treatment benefit despite side effects
Source: Qual Life Res. 2021 May 31;31(1):135–46. doi: 10.1007/s11136-021-02882-6 (PMC8800875; doi:10.1007/s11136-021-02882-6)
Supplement: Supplementary file 2 — Supplementary file2 (DOCX 43 kb) [file 11136_2021_2882_MOESM2_ESM.docx]

Appendix II

ARCTIC Study: Independent Ethics Committees/Institutional Review Boards

Australia

- SMHS Research Ethics and Governance Unit, Palmyra DC, WA, Australia

Belgium

- Comité d’Ethique Hospitalo, Facultaire Universitaire de Liège, Liège, Belgium

Bulgaria

- Ethics Committee for Multicenter Trials, Sofia, Bulgaria

Canada

- Horizon Health Network Research Ethics Board, Saint John, N.B., Canada

Chile

- Comité de Evaluación Ética Científica Servicio de Salud Araucanía Sur Temuco, Chile
- Comité de Ética de la Investigación Servicio de Salud Metropolitano Norte Calle San José, Santiago, Chile
- Comité de Ética Científica, Clínica Santa María Av. Bellavista, Providencia, Santiago, Chile

Czech Republic

- Eticka komise Krajska nemocnice Liberec, Czech Republic

France

- Comité de Protection des Personnes Ile de France V, Paris, France

Germany

- Ethik-Kommission des Landes Sachsen-Anhalt, Sachsen Anhalt, Germany

United Kingdom

- South Central - Hampshire A Research Ethics Committee, Bristol, United Kingdom

Greece

- National Ethics Committee (Greece), Cholargos, Greece

Hong Kong

- Institutional Review Board of the University of Hong Kong/Hospital Authority Hong Kong West Cluster, Hong Kong
- Research Ethics Committee (Kowloon Central/Kowloon East) Queen Elizabeth Hospital, Kowloon, Hong Kong
- Kowloon West Cluster Clinical Research Ethics Committee, Kowloon, Hong Kong

Hungary

- Medical Research Council - Ethics Committee For Clinical Pharmacology, Budapest, Hungary
- Egeszsegugyi Tudomanyos Tanacs Klinikai Farmakologiai Etikai Bizottsaga Arany, Budapest, Hungary

Israel

- Helsinki Committee Hospital Management Tel Hashomer, Israel

Italy

- Comitato Etico Indipendente della Fondazione IRCCS Istituto Nazionale dei Tumori di Milano, Milano, Italy

Japan

- Osaka International Cancer Institute IRB, Osaka, Japan
- NHO Kinki-Chuo Chest Medical Center IRB, Osaka, Japan
- Shizuoka Cancer Center IRB, Sunto-gun, Shizuoka-Ken, Japan
- Kansai Medical University Hospital IRB, Hirakata-shi, Osaka-Fu, Japan
- Miyagi Cancer Center IRB, Natori, Miyagi, Japan
- Saitama Medical University International Medical Center IRB, Hidaka, Saitama, Japan
- Osaka Habikino Medical Center IRB, Habikino, Osaka, Japan
- Hiroshima City Hiroshima Citizens Hospital IRB, Hiroshima-shi, Hiroshima-Ken, Japan
- Kanazawa University Hospital IRB, Kanazawa, Ishikawa, Japan
- NHO Nagoya Medical Center IRB, Nagoya, Aichi, Japan
- Kobe City Hospital Organization Kobe City Medical Center General Hospital IRB, Kobe, Hyogo, Japan
- National Center for Global Health and Medicine Hospital IRB, Shinjuku-ku, Tokyo-To, Japan
- NHO Shikoku Cancer Center IRB, Matsuyama, Ehime, Japan
- Wakayama Medical University Hospital IRB, Wakayama, Japan
- Yokohama City University Medical Center IRB, Yokohama, Kanagawa, Japan
- Nagoya University Hospital IRB, Nagoya, Aichi, Japan
- NHO Hirosaki National Hospital IRB, Hirosaki, Aomori, Japan
- Okayama University Hospital IRB, Okayama-shi, Okayama-Ken, Japan
- Aichi Cancer Center Hospital IRB, Nagoya, Aichi, Japan
- Tokyo Medical University Hospital IRB, Shinjuku-ku, Tokyo, Japan
- Kouseikai Sendai Kousei Hospital IRB, Sendai, Miyagi, Japan
- Osaka Medical College Hospital IRB, Takatsuki, Osaka, Japan
- Hokkaido Cancer Center IRB, Sapporo, Hokkaido, Japan
- Yokohama Municipal Citizen's Hospital IRB, Yokohama Kanagawa, Japan
- Kurume University Hospital IRB, Kurume-shi, Fukuoka-Ken, Japan
- Kindai University Hospital IRB, Osakasayama, Osaka, Japan
- Hokkaido University Hospital IRB, Sapporo-shi, Hokkaido, Japan
- Kyushu University Hospital IRB, Japan

Netherlands

- Stichting Beoordeling Ethiek Biomedisch Onderzoek, Assen, KV, Netherlands

Poland

- Komisja Bioetyczna przy Centrum Onkologii, Warszawa, Poland

Korea

- IRB of Chungbuk National University Hospital, Cheongju-si, Chungcheongbuk-do, Korea
- IRB of Samsung Medical Center, Gangnam-gu, Seoul, Korea
- IRB of Asan Medical Center, Songpa-gu, Seoul, Korea
- Korea University Anam Hospital, Seongbuk-Gu, Seoul, Korea
- Dong-A University Hospital, Seo-gu, Busan-si, Korea
- Gachon University Gil Medical Center, Namdong-gu, Incheon-si, Korea
- Chonnam National University Hwasun Hospital, Hwasun-Gun, Jeollanam-do, Korea
- Seoul National University Bundang Hospital, Seongnam-si, Gyeonggi-do, Korea

Romania

- The National Bioethics Committee for Medicine and Medical Devices, Bucuresti, Romania

Russia

- Ethical Council at the MoH of RF, Moscow, Russia
- LEC of FSBI "Scientific Research Institute of Oncology n. a. N. N. Petrov", Saint-Petersburg, Russia

Serbia

- Ethics Committee of Clinical Center Bezanijska Kosa, Belgrade, Serbia
- Ethics Committee of Clinical Center Kragujevac, Kragujevac, Serbia
- Ethics Committee of Institute of Oncology and Radiology of Serbia, Belgrade, Serbia
- Ethics Committee of Clinical Center Nis, Nis, Serbia
- Ethics Committee of Institute for Pulmonary Diseases of Vojvodina, Sremska Kamenica, Serbia

Singapore

- National Healthcare Group Domain Specific Review Board, Nexus@one-north, Singapore

Spain

- CEIC Grupo HM, Hospital Universitario Madrid Montepríncipe, Madrid, Spain

Republic of China

- Central Institutional Review Board, Taipei, Taiwan, Republic of China

Thailand

- Central Research Ethics Committee (CREC), National Research Council of Thailand, Bangkok, Thailand

United States of America

- Copernicus Group IRB, Cary, North Carolina, USA
- WIRB, Puyallup, WA, USA
- Washington University in St. Louis IRB, St. Louis, MO, USA
- BRANY IRB, Lake Success, New York, USA
- Sharp Healthcare IRB, San Diego, CA, USA
- UCSD Human Research Protection Program, La Jolla, CA, USA
- Rush University Medical Center, Chicago, IL, USA
